# Evidence synthesis of medical cannabis research: current challenges and opportunities

**DOI:** 10.1007/s00406-024-01893-x

**Published:** 2024-11-09

**Authors:** Ben Senator, Mafalda Pardal, Liesbeth Vandam

**Affiliations:** 1 RAND Corporation and Pardee RAND Graduate School, Santa Monica, California USA; 2 RAND Europe, Brussels, Belgium; 3 EUDA, Lisbon, Portugal

**Keywords:** Cannabis, Cannabinoids, Medical cannabis, Evidence synthesis, Systematic review, Meta-analysis

## Abstract

As a wide group of medicines, the effectiveness and safety of ‘medical cannabis’ products is likely to vary in relation to product-specific dimensions such as potency, dosage, route of administration, and cannabinoid composition. Systematic reviews can perform a crucial role in analysing and synthesising the outcomes of medical cannabis interventions found in empirical research. We analysed 23 contemporary systematic reviews on the effectiveness and safety of medical cannabis to discern the extent to which this body of work aimed to capture, and ultimately captured, the differing outcomes of medical cannabis products by product-specific dimensions of treatment. We further highlighted the methodological reasons given by authors for an inability to describe this granular level of information. We found that a minority of systematic reviews explicitly aimed to perform a subgroup analysis to determine differences in treatment outcomes by product-specific dimensions of medical cannabis, with even fewer subsequently doing so. Authors’ stated reasons for this concerned either overly large or overly small levels of variation in the characteristics, compositions, and administrations of medical cannabis products used, rendering subgroup analyses methodologically inappropriate or inapplicable. Furthering systematic reviews’ abilities to capture granular information on medical cannabis treatment outcomes in relation to product-specific dimensions of treatments will require further standardisation of treatments in empirical studies.

## Introduction

Many countries worldwide have reformed laws to allow patients to access medical cannabis products,^1^ including most U.S. states and Canadian provinces, a number of EU Member States such as the Netherlands, Belgium, France, Germany and other international jurisdictions such as Australia and Israel [1, 2]. These regulatory frameworks foresee different approaches concerning access to medical cannabis products, the type of products available, and under which circumstances they can be accessed (e.g. who is authorised to prescribe them, for which conditions), among other aspects. Regulations regarding medical cannabis primarily cover the types of products available to patients and the mechanism by which they may access them [3, 4]. Indeed, medical cannabis products exist in a variety of forms, including plant-based products (made directly from the cannabis plant), plant-derived cannabinoids (compounds extracted directly from the cannabis plant, such as the psychoactive cannabinoid delta-9-tetrahydrocannabinol (THC) and non-psychoactive cannabinoid cannabidiol (CBD)), and synthetic preparations (chemically manufactured products, rather than extracted). Medical cannabis products may have undergone a marketing authorization process to be recognised as medicines, typically following several clinical and non-clinical trials as well as compliance with other requirements (e.g. quality assurance) that facilitate a robust understanding of a product’s effectiveness and safety profile [11]. In addition, in other cases, products may have followed simplified authorization processes [11]. Across EU Member States, commonly market-authorised medical cannabis products are pharmaceutical preparations, including synthetic THC products, such as dronabinol and nabilone, and products that include cannabis-derived THC and/or CBD, such as Sativex (also known as nabiximols) and Epidyolex (purified CBD oral oil) [3, 5]. Although the different EU Member States have unstandardised regulations for medical cannabis products’ accessibility, the licensing and market authorization process typically involves a medical cannabis product passing through several clinical trials [6, 7]. Following successful clinical trials, most EU Member States have now licensed some form of a pharmaceutical preparation of medical cannabis (either synthetic or plant-derived), and in these jurisdictions physicians can choose to prescribe the product to patients, subject to any further national or subnational regulations [5].

Internationally, there is a diversity of access mechanisms to medical cannabis products. Compassionate access or exceptional use schemes have been established in some EU Member States and other non-EU countries, whereby a patient – typically with a serious condition that is unresponsive to existing treatments – can have exceptional approval from the pharmaceutical regulator to access an unlicensed medical cannabis product [6, 7].^2^ This approach can be found in Australia, whereby unlicensed medical cannabis products can be prescribed by medical practitioners under a Special Access Scheme or Authorised Prescriber Scheme [8]. Whilst no plant-based herbal medical cannabis products have received a formal marketing authorization across the EU, the Netherlands, Czech Republic, Denmark, and Italy have established a system for patient access to medicinal-grade herbal cannabis products [9]. In the U.S. context, although cannabis with a THC level of greater than 0.3% is listed as a Schedule I substance at federal level and as such prohibited from consumption, possession, manufacture, and sale, most states have reformed law to permit differing levels of accessibility to medical cannabis products, with substantial variation in regulatory approaches at the state level [10, 11].^3^

Where permitted, medical cannabis products are used to treat a range of conditions. Most commonly, medical cannabis products are used in pain management, but are also used in the treatment of numerous other conditions, such as mental health conditions, dementia, sleep disorders, and the alleviation of nausea [12].

### Current knowledge needs and gaps regarding medical cannabis

With these regulations in place in many countries inside and outside of the EU, a number of stakeholder groups will require rigorous evidence on the use of medical cannabis from a clinical practice perspective and a solid understanding of regulatory frameworks related to patient access.

Evidence suggests the number of medical cannabis users is increasing [13, 14]; hence, there will be a growing number of individuals and patients who are interested in exploring whether medical cannabis products – both those with and without a marketing authorization – could be used for their conditions. In turn, healthcare practitioners – including attending physicians and general practitioners (i.e. primary care physicians) – will need to be prepared to navigate clinical care decisions and discussions about medical cannabis products with patients [15, 16]. This pressures the development of evidence-informed clinical guidance, which will allow both patients and practitioners to be informed when considering medical cannabis products for specific conditions [17]. From a wider perspective, strong evidence will be required by policymakers, insurance providers, and healthcare organizations who wish to understand the risks and benefits of medical cannabis products, and accordingly adapt their respective policies – such as regulatory approaches – to meet their objectives [18].

Some medical cannabis products have received marketing authorizations in some EU Member States, such as Sativex and Epidyolex. For these products, which will have only been approved following extensive clinical trials on their effectiveness and safety, clinical guidance for healthcare practitioners has been drafted and disseminated under the marketing authorization process, with such guidance developed by expert clinician groups and the product manufacturer. The development of guidance for healthcare practitioners has also been seen in jurisdictions that permit access to some medical cannabis products outside of a formal marketing authorization process, such as in Australia and the U.S [8, 17].

Despite the development of guidance for prescription across both market-authorized and medical cannabis products without this type of approval, and recent growth in the quantity of evidence from clinical and observational studies on the benefits and harms of medical cannabis, literature suggests that stakeholders frequently lack actionable knowledge on cannabis and cannabinoids for medical use. Surveys from multiple countries have found healthcare practitioners and providers to be often uncertain when discussing and deciding to prescribe medical cannabis products with patients, suggesting that physicians commonly perceive a lack of clinical evidence on the effectiveness and safety of cannabis and cannabinoids for medical use [19–23].

The development of evidence-based resources will need to consider the benefits and harms of medical cannabis products for the treatment of specific conditions. But, just as importantly, such resources will need to consider how those benefits and harms may vary by the product-specific characteristics of medical cannabis [17]. In contemporary reviews of the evidence base, it has been noted that strong evidence is currently lacking on the relationship between the impacts of medical cannabis on patients and the product-specific information of the treatment used, including the route and frequency of administration, type, dosage, and potency of medical cannabis used [9, 17, 24]. As a variety of different forms of prescribable medical cannabis products exist, the investigation of treatment impacts by product-specific dimensions of medical cannabis will be a critical research avenue to improve the evidence base [18].

### How systematic reviews can help

As one of the highest hierarchical levels of evidence in evidence-based medical decision-making [25, 26], systematic reviews can perform a key role in synthesizing individual studies on medical cannabis to bridge the gap between evidence and stakeholder knowledge. Indeed, a number of systematic reviews on the effictiveness and safety of medical cannabis have been conducted to date, with a recent scoping review of systematic reviews on the benefits and harms of medical cannabis for the treatment of any condition by identifying 72 systematic reviews eligible for inclusion [12]. These reviews are often conducted, commissioned, or used by policymaking, as well as by policy-influencing bodies and institutions, including the European Union Drugs Agency [6] and the National Academies of Sciences [18], with aims to inform decision making and provide expert advice to relevant stakeholders.

Furthermore, systematic reviews may further be able to provide a granular level of evidence through a subgroup analysis. Subgroup analyses allow systematic reviewers to investigate study heterogeneity by conducting their analysis on a subset of studies that share a particular characteristic; in clinical and medical evidence synthesis, this will typically be by the condition of the patient or the type of treatment used [27]. Considering the need to gain further knowledge regarding the treatment impacts of medical cannabis by product-specific dimensions, the use of subgroup analyses in systematic reviews may provide a strong opportunity to improve the medical cannabis evidence base.

However, in a recent review of systematic reviews (Jan 2018-Mar 2022) on the effectiveness and safety of medical cannabis products for any condition conducted for the EMCDDA^5^ [28], we (BS, MP, and co-authors) noted that the majority of the 24 included systematic reviews did not provide sufficient insight into the benefits and harms by route and frequency of administration, type, potency, and dosage. Although analysis by condition was consistent across reviews, we observed that some reviews did not give appropriate explicit attention to the heterogenous dimensions of medical cannabis and cannabinoid-based treatments in relation to its effectiveness and safety.

### Goals of this study

Systematic reviews can be a helpful tool in providing a high level of evidence into medical use of cannabis. In our analysis, we investigate the extent to which contemporary systematic reviews focusing on the effectiveness and safety of medical cannabis and cannabinoid treatments fully capture the granularity stakeholders need in relation to four product-specific dimensions of treatment:

1. The type of product or cannabinoid composition of product, i.e. the specific form of cannabis in reference to a generic (e.g., whole plant products) or brand name of a medical cannabis product (e.g. Sativex, Epidyolex), or in reference to the chemical and cannabinoid composition of the treatment product.
2. The route of administration, i.e. the method in which the cannabis product is introduced to the body (e.g. inhalation, oral, topical).
3. The frequency or length or administration, i.e. the number of times the cannabis treatment is administered to the individual over a regular treatment period (e.g. twice daily, once per week) and the overall length of treatment (e.g. two weeks, six months).
4. The potency or dosage of the medical cannabis product, i.e. the specific amount or concentration of cannabinoids in a treatment product (e.g., percentage or milligrams of THC/CBD), and the amount of the product consumed in one treatment administration session (e.g., number of capsules, quantity of herbal cannabis).

We do so by drawing on a sample of systematic reviews gathered in a previous study (Pardal et al. forthcoming; see Sect. 2.1), and analyse them in relation to two main research questions:

1. To what extent did those systematic reviews aim to analyse, and thereafter conduct a subgroup analysis of, the impacts of medical cannabis by the four aforementioned product-specific dimensions?
2. In cases where a subgroup analysis by product-specific dimension of medical cannabis was not conducted, what were the reasons put forward by the authors?

We include this latter research question to facilitate an understanding of the enablers and barriers to conducting subgroup analyses within systematic reviews in this field. By extracting this information from reviews and highlighting common enablers and barriers across the systematic review evidence base, these insights may allow for greater consideration of the feasibility of a subgroup analysis by researchers undertaking systematic reviews of medical cannabis evidence at the protocol stage. Such considerations will be important to continue to develop the knowledge base on cannabis used for medical purposes.

## Methods

### An overview of our approach in Pardal et al. (forthcoming)

The systematic reviews we analyse here were searched for, identified, screened for eligibility, and included for full review in accordance with the processes undertaken in the authors’ previous study [28]. In Pardal et al. [28], we carried out a rapid review of systematic reviews, published between January 2018 and March 2022, that captured evidence on the effectiveness and safety of medical cannabis products for the treatment of symptoms of various medical conditions. As with our previous terminology that takes ‘medical cannabis’ in its broadest sense, the term ‘cannabis and cannabinoids for medical use’ is used to denote the full range of herbal cannabis products, extracts, and synthetic cannabis products used for medical purposes, including those with and without marketing authorizations. We refer to ‘effectiveness’ as an intervention’s capacity to produce the desired effect under typical circumstances [29]. The use of medical cannabis products for the treatment of an indication is taken as our intervention. We take ‘safety’ to denote the extent to which the intervention avoids, prevents, or mitigates harms that arise from the administration of the intervention.

A ‘review of systematic reviews’ or ‘review of reviews’ refers to a methodological approach to literature collation and synthesis in which only systematic reviews are included in the review. The prefix ‘rapid’ indicates that the review takes a narrower and simplified approach to literature collation and synthesis in comparison to a traditional systematic review, whilst maintaining rigor and explicitness in terms of the methodological steps undertaken. Our review was rapid in the sense that it only aimed to capture systematic reviews published during a specific and short time-frame (January 2018 - March 2022), and limited the sources selected for full-text review by first assessing all inclusion-eligible systematic reviews by the following inclusion criteria: English language; published in 2018 or later as a peer-reviewed journal article; is a systematic review or meta-analysis; focuses on effectiveness and safety of medical cannabis for human use for any indication or therapeutic area; includes information on review methodology and quality of evidence assessment.

Four literature databases and literature search tools were selected for searching based on their likely relevance to the research topic and potential to capture all relevant systematic reviews: Cochrane Database of Systematic Reviews; Embase; PubMed; and Web of Science. Searches were conducted between February and March 2022, after an earlier piloting of the search terms. All records identified across all databases were loaded into EndNote for subsequent processing after the searches were run. The searches yielded an initial *n* = 395 potential systematic reviews, prior to removal of duplicates and screening. After removing duplicates (*n* = 163) and sources captured prior to 2018 (*n* = 42), *n* = 190 systematic reviews remained and were screened against inclusion and exclusion criteria. The screening process resulted with a yield of *n* = 42 inclusion-eligible systematic reviews, and a final *n* = 24 systematic reviews selected for full text review.^4^

### Approach for the present analysis

The current analysis is based on the systematic reviews identified in Pardal et al. [28], which as described above resulted in *n* = 24 systematic reviews. The findings from those reviews concerning the effectiveness and safety of cannabis for medical use across a range of conditions is captured in that publication [28], and we do not repeat that here. Rather, in line with the research goals outlined above, we focus on the extent to which reviews aimed to capture the impacts of medical cannabis interventions in relation to the product-specific dimensions of the intervention itself. For the present analysis, we additionally do not focus on reviews of reviews. As such, after removing one review of reviews [30] from the initially identified sample, *n* = 23 systematic reviews were integrated in the present analysis.

To that end, we first examined the types of data that were extracted from the included studies in these systematic reviews. Specifically, we reviewed whether each systematic review collected data on the product-specific dimensions of interest regarding the medical cannabis intervention they studied: (1) the type of product or cannabinoid composition of product; (2) the route of administration; (3) the frequency or length or administration; and (4) the potency or dosage of the medical cannabis product. Our definitions for these product-specific dimensions are given in Sect. 1.3. For each dimension, we indicated on the data extraction spreadsheet whether the systematic review explicitly did or did not extract this data, or mark if unclear if the systematic review did not give any indication as to whether data for a particular product-specific dimension was extracted.

We additionally scanned the full text, appendices, and any supplementary materials of each systematic review to note whether the systematic review explicitly aimed for, and conducted, a subgroup analysis by a product-specific dimension of interest. We considered a systematic review to have performed a subgroup analysis by any of the dimensions of interest if the review conducted any of the following: (1) performed a quantitative meta-analysis stratified by the dimension(s) of interest; (2) utilized a random-effects model in meta-analysis in which the effect size of treatment was allowed to vary by covariates that captured a product-specific dimension of medical cannabis; or (3) performed a qualitative/narrative synthesis of evidence that was clearly delineated by the dimension(s) of interest. Finally, we noted the explicitly stated reason that a systematic review gave for not conducting a subgroup analysis by a product-specific dimension of medical cannabis. These were initially lifted verbatim from the full text of the systematic review, before being thematically categorized by the research team.

As the systematic reviews analysed in this paper were taken as a sample from the broader base of scientific literature on medical cannabis for the purpose of our analysis, we cannot claim that our included reviews are comprehensive of all existing medical cannabis research. To this extent, the findings of this study regarding the barriers and opportunities in synthesising medical cannabis research may not generalize to all systematic reviews in the knowledge field. Rather, in taking a sample of systematic reviews, we conduct an exploratory analysis of some of the existing barriers and opportunities in systematic review publications that arise in medical cannabis research. Readers should note that other reviews not captured in this study may have encountered barriers and opportunities not highlighted in our findings.

## Findings

The 23 included systematic reviews covered a substantial range of diverse medical cannabis products for a range of conditions, reflecting previous discussions on the wide variety of available licensed and unlicensed medical cannabis products available to patients. Whilst the majority of reviews analysed the effects of pharmaceutical preparations, such as nabilone, dronabinol, and synthetic THC/CBD/THC: CBD isolates, it was not uncommon for studies to additionally use herbal cannabis preparations as intervention. The effects of herbal cannabis preparations were captured in reviews by Stockings, Zagic [31], Mücke, Weier [32], and Black, Stockings [33], and the review conducted by Elliott, DeJean [34] included studies that examined cannabis products used with medicinal intent obtained from illicit suppliers.

Reviews captured a range of study designs, including randomized controlled trials and non-randomized observational studies. A large number of conditions were studied across all the reviews, including Alzheimer’s and dementia, anorexia nervosa, anxiety disorders, cancer-associated cachexia, cannabis withdrawal, epilepsy, inflammatory bowel disease, multiple sclerosis, pain, posttraumatic stress disorder, and spinal cord injury. An overview of the 23 systematic reviews we analysed is presented in Table 1.

**Table 1:** Overview of included systematic reviews

Systematic review	Review period	Number of studies included	Study designs from reviewed studies	Conditions studied	Medical cannabis products used ^a^
Bahji, Meyyappan [35]	?-2019	11	RCTs = 6 Open-label = 5 Cohort = 3	Social anxiety disorder Post-traumatic stress disorder Generalized anxiety disorder	Nabilone CBD THC
Black, Stockings [33]	1980–2018	83	RCTs = 50 Open-label trials = 15 Prospective cohort = 9 Cross-sectional/retrospective = 14 Chart review = 6 Case studies = 17	Multiple disorders	Cannabis sativa Nabiximols Dronabinol Nabilone THC extracts CBD extracts THC: CBD extract
Desmarais, Smiddy [36]	?-2019	3	RCTs = 3	Inflammatory bowel disease Ulcerative colitis Crohn’s disease	CBD THC
Elliott, DeJean [34]	?-2019	123	Published reports = 41 Conference abstracts = 50 Trial registration records = 32	Multiple forms of epilepsy	CBD CBD: THC Products from illicit cannabis suppliers Home-made extracts Artisanal products
Fisher, Moore [37]	?-2019	36	RCTs = 36 (31 studies included in quantitative synthesis, meta-analysis)	Multiple forms of pain	Nabiximols THC Cannabis Palmitoylethanolamide (PEA) Fatty acid amide hydrolase inhibitors (FAAH) Dronabinol Nabilone Cannabis receptor agonist THC congener receptor agonist
Gazendam, Nucci [38]	?-2019	6	RCTs = 6	Acute pain	Nabilone AZD1940 Synthetic CB2-selective agonist Plant-based cannabis oil extract Levonantradol
Hoch, Niemann [39]	2006–2018	18	SRs = 4 (of 11 RCTs) RCTs = 14	Multiple disorders	Dronabinol Nabiximols Nabilone Rimonabant Drinabant THC CBD
Kurlyandchik, Tiralongo [40]	?-?	10	RCTs = 3 Observational studies = 6 Crossover study = 1	Fibromyalgia	Bedrocan Bediol Bedrolite Nabilone Dronabinol
Larsen and Shahinas [41]	2000–2019	25	RCTs = 20 NRCTs = 2 Observational studies = 3	Multiple disorders	CBD
Lattanzi, Brigo [42]	?-2018	2	RCTs = 2	Multiple forms of epilepsy	CBD
Mücke, Weier [32]	?-2017	9	RCTs = 9	Multiple disorders	Dronabinol Nabiximols THC: CBD Herbal cannabis
Nabata, Tse [43]	?-2020	34	Experimental studies = 8 Observational studies = 26	Spinal Cord Injuries	Dronabinol Nabilone CBD THC THC: CBD
Price, Charlot [44]	?-2020	4	RCTs = 3 Prospective cohort study = 1	Multiple forms of neck and back pain	THC Dronabinol Nabilone
Rehman, Saini [45]	?-2020	11	RCT = 1 Observational studies = 10	PTSD	Nabilone CBD THC
Rosager, Møller [46]	?-2020	4	RCTs = 4	Anorexia nervosa	Dronabinol THC
Ruthirakuhan, Lanctôt [47]	?-2018	6	RCTs = 5 Open-label = 1	Alzheimer’s disease	THC Dronabinol Nabilone
Sainsbury, Bloxham [48]	?-2021	17	RCTs = 17	Chronic neuropathic pain (NP)	THC-CBD: Oromucosal spray Sativex THC CBDV CT-3 Synthetic cannabinoid
Simon, Baldwin [49]	?-2020	10	RCTs = 4 NRSI = 6	Cachexia	THC CBD Dronabinol Nabilone
Stella, Valiengo [50]	1990–2021	15	Clinical trials = 5 Open studies = 3 Case/series reports = 7	Dementia	THC CBD THC-CBD Nabilone Dronabinol
Stockings, Zagic [31]	?-2017	35	Observational studies = 30 RCTs = 6	Epilepsy	CBD CBD: THC extract Cannabis sativa Dronabinol
Vivace, Sanders [51]	?-2020	13	RCTs = 4 Cohort studies = 2 Cross-over = 3 Dose escalation trial = 1 Case series = 3	Multiple forms of pain	Nabilone THC CBD THC: CBD Dronabinol
Wang, Hong [52]	?-2021	28	RCTs = 28	Multiple forms of pain	CBD CBDV PEA THC THC: CBD
Werneck, Kortas [53]	?-2017	10	RCTs = 6 NRCTs = 4	Cannabis withdrawal symptoms	Dronabinol Nabilone Nabiximols

^a^We refer to the cannabis products as cited in the systematic reviews; in some cases, further specification of products was lacking

The vast majority of the systematic reviews we analysed extracted data on all product-specific dimensions of cannabis and cannabinoid treatments that were of interest to this study, as highlighted in Table 2. Out of the 23 systematic reviews we analysed, 22 extracted data regarding the route of administration and the type of product or cannabinoid composition of the product. A total of 21 systematic reviews extracted data regarding the potency or dosage of the medical cannabis product, and the frequency or length of administration of treatment. However, many reviews did not specify a precise generic or brand name for the medical cannabis product(s) they analysed.

**Table 2:** Included systematic reviews, by whether data was extracted regarding a product-specific dimension of interest

Systematic review	Type of product / cannabinoid composition	Route of administration	Frequency / length of administration	Potency / dosage
Bahji, Meyyappan [35]	+	+	+	+
Black, Stockings [33]	+	+	+	+
Desmarais, Smiddy [36]	/	/	/	/
Elliott, DeJean [34]	+	+	+	/
Fisher, Moore [37]	+	+	+	+
Gazendam, Nucci [38]	+	+	+	+
Hoch, Niemann [39]	+	+	+	+
Kurlyandchik, Tiralongo [40]	+	+	+	+
Larsen and Shahinas [41]	+	+	+	+
Lattanzi, Brigo [42]	+	+	+	+
Mücke, Weier [32]	+	+	+	+
Nabata, Tse [43]	+	+	+	+
Price, Charlot [44]	+	+	+	+
Rehman, Saini [45]	+	+	/	+
Rosager, Møller [46]	+	+	+	+
Ruthirakuhan, Lanctôt [47]	+	+	+	+
Sainsbury, Bloxham [48]	+	+	+	+
Simon, Baldwin [49]	+	+	+	+
Stella, Valiengo [50]	+	+	+	+
Stockings, Zagic [31]	+	+	+	+
Vivace, Sanders [51]	+	+	+	+
Wang, Hong [52]	+	+	+	+
Werneck, Kortas [53]	+	+	+	+
Total number of studies that extracted data (max 23)	**22**	**22**	**21**	**21**

+ extracted, / unclear if extracted, - not extracted

Some systematic reviews, such as Stella, Valiengo [50], did not explicitly clarify the information they extracted. In these instances, we examined results, tables, figures, and other supplementary materials to be able to infer that all information regarding the product-specific dimensions of interest were extracted from individual studies. Other reviews, such as Gazendam, Nucci [38], detailed their data extraction process with references to the data fields they captured. After examining all reviews, only 3 reviews [34, 36, 45] remained unclear as whether they extracted data on some or all of the product-specific dimensions of interest. There were no instances in which it appeared certain that a systematic review did not extract data on a product-specific dimension of treatment.

Table 3 presents the findings from the second section of our analysis, where we analysed whether the 23 systematic reviews aimed to conduct, and ultimately conducted, a subgroup analysis by a product-specific dimension of medical cannabis. The most commonly aimed-for subgroup analysis was for the type of product or cannabinoid composition of the product, with 7 out of the 23 reviews explicitly aiming to conduct this analysis. Route of administration and the potency or dosage of treatment were the next most commonly aimed-for subgroup analyses, with 5 reviews out of the 23 aiming to conduct the subgroup analysis for each product-specific dimension. A subgroup analysis by the frequency or length of treatment administration was the least frequently aimed-for subgroup analysis, with 4 out of 23 reviews aiming to conduct the analysis.

**Table 3:** Included systematic reviews by whether a subgroup analysis was aimed for, and whether a subgroup analysis was conducted, by product-specific dimension

	Type of product / cannabinoid composition		Route of administration		Frequency / length of administration		Potency / dosage	
**Systematic review**	Subgroup analysis aim	Subgroup analysis conducted	Subgroup analysis aim	Subgroup analysis conducted	Subgroup analysis aim	Subgroup analysis conducted	Subgroup analysis aim	Subgroup analysis conducted
Bahji, Meyyappan [35]	+	+	-	-	+	+	+	+
Black, Stockings [33]	+	+	-	-	-	-	-	-
Desmarais, Smiddy [36]	-	-	-	-	-	-	-	-
Elliott, DeJean [34]	-	-	-	-	-	-	-	-
Fisher, Moore [37]	+	+	+	-	+	-	+	-
Gazendam, Nucci [38]	+	-	+	+	-	-	-	-
Hoch, Niemann [39]	-	-	-	-	-	-	-	-
Kurlyandchik, Tiralongo [40]	-	-	-	-	-	-	-	-
Larsen and Shahinas [41]	-	-	+	-	+	-	+	-
Lattanzi, Brigo [42]	-	-	-	-	-	-	+	-
Mücke, Weier [32]	-	-	+	-	-	-	+	-
Nabata, Tse [43]	-	-	-	-	-	-	-	-
Price, Charlot [44]	-	-	-	-	-	-	-	-
Rehman, Saini [45]	-	-	-	-	-	-	-	-
Rosager, Møller [46]	-	-	-	-	-	-	-	-
Ruthirakuhan, Lanctôt [47]	+	+	-	-	+	+	-	-
Sainsbury, Bloxham [48]	+	+	-	-	-	-	-	-
Simon, Baldwin [49]	-	-	-	-	-	-	-	-
Stella, Valiengo [50]	-	-	-	-	-	-	-	-
Stockings, Zagic [51]	-	-	-	-	-	-	-	-
Vivace, Sanders [51]	-	-	-	-	-	-	-	-
Wang, Hong [52]	+	+	+	+	-	-	-	-
Werneck, Kortas [53]	-	-	-	-	-	-	-	-
Total number of studies that aimed for or conducted a subgroup analysis	7	6	5	2	4	2	5	1

+ aimed for/conducted, / unclear if aimed for/conducted, - not aimed for/not conducted

Of the 7 reviews that aimed to conduct a subgroup analysis by type of product or cannabinoid composition of the product, 6 were ultimately able to conduct the analysis. Fisher, Moore [37], for example, analyzed the change score data after stratifying by the type of product used. Sainsbury, Bloxham [48] found heterogeneity of cannabis products used across studies, and stratified their statistical analysis across THC/CBD, CBD, cannabidivarin (CBDV), and synthetic cannabis. A similar approach was used by Wang, Hong [52], who examined the differences in treatment effects by THC alone, THC and CBD, and CBD alone, as well as PEA. However, far fewer were able to conduct the intended subgroup analysis for the other identified product-specific dimensions of interest. For instance, of the 5 reviews that aimed to conduct a subgroup analysis by potency or dosage of product, only Bahji, Meyyappan [35] were able to do so.

To further understand the reasons driving this low frequency of conducted subgroup analyses, Table 4 presents the systematic reviews we reviewed by two researcher-categorized fields: the reviews’ methodological approaches to subgroup analyses (if applicable), and the review’s author-stated reasons for not ultimately conducting a subgroup analysis (if stated). Of the systematic reviews that were able to perform a subgroup analysis, all but one review [35] utilized a stratified meta-analysis, whereby studies were stratified by the dimension of interest with treatment outcomes quantitatively synthesized within each stratification. One review [35] gave insight into the changes in treatment effect sizes by product-specific dimensions by studying the direction and statistical significance of covariates in their meta-regression analysis, finding that there were no differences in the effect sizes of treatments by treatment dosages.

**Table 4:** Included systematic reviews with researcher-categorized methodological approach to subgroup analysis, and researcher-categorized reason for inability to conduct subgroup analysis by (additional) dimensions

Systematic review	Subgroup analysis conducted by at least one dimension?	Categorized methodological approach to subgroup analysis by dimension	Categorized reason for inability to conduct subgroup analysis by (additional) dimensions
Bahji, Meyyappan [35]	Yes	Heterogeneity-causing covariate analysis	N/A
Black, Stockings [33]	Yes	Stratified meta-analysis	Substantive overall study heterogeneity
Desmarais, Smiddy [36]	No	N/A	None given
Elliott, DeJean [34]	No	N/A	Substantive intervention heterogeneity
Fisher, Moore [37]	Yes	Stratified meta-analysis	Substantive overall study homogeneity
Gazendam, Nucci [38]	Yes	Stratified meta-analysis	Substantive intervention homogeneity
Hoch, Niemann [39]	No	N/A	Substantive measurement heterogeneity
Kurlyandchik, Tiralongo [40]	No	N/A	None given
Larsen and Shahinas [41]	No	N/A	Substantive measurement heterogeneity
Lattanzi, Brigo [42]	No	N/A	Substantive intervention homogeneity
Mücke, Weier [32]	No	N/A	Too few overall studies
Nabata, Tse [43]	No	N/A	Substantive overall study heterogeneity
Price, Charlot [44]	No	N/A	None given
Rehman, Saini [45]	No	N/A	Substantive overall study heterogeneity
Rosager, Møller [46]	No	N/A	None given
Ruthirakuhan, Lanctôt [47]	Yes	Stratified meta-analysis	N/A
Sainsbury, Bloxham [48]	Yes	Stratified meta-analysis	N/A
Simon, Baldwin [49]	No	N/A	None given
Stella, Valiengo [50]	No	N/A	None given
Stockings, Zagic [31]	No	N/A	None given
Vivace, Sanders [51]	No	N/A	None given
Wang, Hong [52]	Yes	Stratified meta-analysis	N/A
Werneck, Kortas [53]	No	N/A	Substantive overall study heterogeneity

Regarding the reasons given by review authors on the inability to conduct a subgroup analysis by a product-specific dimension of cannabis and cannabinoid treatment, 7 reviews gave reference to substantive heterogeneity across studies as a barrier for cross-study comparisons. As detailed below, study heterogeneity refers to the situation in which a large amount of variation in study characteristics – such as the type of intervention and measurement of outcomes – renders a subgroup analysis statistically impossible.

Some reviews were specific to the source of heterogeneity. Werneck, Kortas [53] were unable to pool data due to a range of heterogeneous study characteristics; the dronabinol and nabiximols dosages varied from 30 to 120 mg, and the frequency of treatment administration of cannabis varied amongst patients from 3.85 g to 22.98 g per week. Vivace, Sanders [51], who reviewed the effect of various pharmaceutical cannabis preparations on pain conditions, found that significant heterogeneity in the type of intervention used in RCTs lead to an inability to perform any form of statistical meta-analysis. Hoch, Niemann [39] were unable to aggregate outcome data due to significant heterogeneity in the measurement of primary outcomes, which similarly made statistical meta-analyses inappropriate. Other reviews were less specific, citing an overall cross-study heterogeneity that limited the extent to which subgroup analyses could be conducted. Many reviews that observed heterogeneity, such as through chi-square tests, mitigated the possibility of treatment effect estimation bias from heterogeneity by utilizing random-effects models in their meta-analytical regression, or by adjusting their synthesis approach to a qualitative review.

Three reviews noted cross-study homogeneity as a reason for an inability to perform a subgroup analysis by a product-specific dimension of cannabis and cannabinoid treatments. Gazendam, Nucci [38], for example, were unable to conduct a subgroup analysis by type of product due to all included studies using the same form of trans-THC as treatment intervention. Similarly, Lattanzi, Brigo [42] were unable to conduct their intended stratified narrative review by daily dose of CBD, as only 2 studies were included and both used the same dosage in their intervention. One review by Mücke, Weier [32] that planned to conduct a subgroup analysis by treatment dosage and route of administration determined that there were too few studies per dimension of interest to make a subgroup analysis feasible, stating that a minimum of 2 would be required.

## Discussion

There is little doubt that interest in the applications of different medical cannabis products has been growing over the previous decades. Since the turn of the century, acceptance of cannabis as a therapeutic substance has rapidly increased: a growing number of jurisdictions have permitted or are considering permitting access to cannabis products for therapeutic purposes; research agendas have been suggested and acknowledged in scientific domains; and patients are increasingly interested in how medical cannabis may be able to treat, or alleviate symptoms of, their own conditions [12, 54]. At the same time, the evidence base on the benefits and harms of medical cannabis is increasing. For some conditions, such as chronic neuropathic pain, the level of evidence is strong enough that the use of cannabis as a medical treatment is well established, and this has been reflected in access to certain medical cannabis products through marketing authorizations in some EU Member States and other pathways in other countries [18, 26, 55].

There are nevertheless important knowledge gaps regarding the potential benefits and harms of cannabis products used in medical treatment. To appropriately consider the potential effectiveness and safety of medical cannabis, it is critical to conceptualise medical cannabis as a far more complex substance than one ‘drug’. As a plant, cannabis contains over 100 known cannabinoids, and a significant number of different products exist, including isolates, extracts, herbal preparations, and synthetic cannabinoids [12, 26]. Research in this area highlights a lack of granular evidence on the interplay between understudied product-specific dimensions, such as the type of cannabis product, routes of administration, and dosages, and the effectiveness and safety of medical cannabis treatments [17, 18].

The present study has highlighted that this granular evidence is often not being captured in systematic reviews of the evidence base on the effectiveness and safety of medical cannabis. Whilst the vast majority of the systematic reviews we analysed extracted data from individual studies on the product-specific dimensions of cannabis of interest to this analysis, far fewer aimed to consider how the product-specific dimension of medical cannabis would impact effectiveness and safety of treatment. Some reviews additionally appeared to underspecify the brand or generic name of the medical cannabis products they included in their analysis. To progress the evidence base of medical cannabis research in this regard, future research would do well to further understand why subgroup analyses by product-specific dimensions of medical cannabis treatments are infrequently highlighted as an objective of a systematic review, despite being an identified area of focus in medical cannabis research agendas.

Amongst those that aimed to conduct a subgroup analysis by a product-specific dimension of treatment, even fewer systematic reviews were successful in utilizing subgroup analyses to explore these interactions. Our research has highlighted that this was primarily due to two explicitly stated reasons. Firstly, some reviews highlighted a lack of variation in the individual studies regarding product-specific dimensions of medical cannabis treatments, such as the type of product used as intervention. Without variation, it is not possible to stratify analyses by a dimension of interest, and a subgroup analysis can subsequently not be conducted. Secondly, multiple reviews stated that there was a substantial amount of heterogeneity in their studies, such as by dosage and frequency of administration, as well as other dimensions beyond product specificities such as outcome measurements. Such variation has indeed been noted by other research in this field, including between independent clinical trials of medical cannabis [4]. If included studies in a systematic review hold little to no alignment by a dimension of interest for a subgroup analysis, then such an analysis will again not be possible.

To progress the evidence base on medical cannabis and its differing effects by product-specific dimensions of treatment, such as type of product, route of administration, dosage, and potency, we make a handful of suggestions justified through our analysis of the 23 systematic reviews discussed. Firstly, we encourage systematic reviews in the field of medical cannabis to be explicit in their stated aims for subgroup analyses, and furthermore explicit in the reasons they did not conduct one, if aimed for. Explicit statements regarding this would allow further insight into the state of the evidence base, and how future review protocols may be developed to maximize the likelihood of providing strong evidence by product-specific dimensions of treatments. Secondly, empirical studies that investigate the effectiveness and safety of medical cannabis should maintain an awareness of other ongoing research, such that where appropriate, evidence-informed alignment and standardisation of medical cannabis interventions and other study characteristics may occur. However, we acknowledge that this is no simple task. There are a range of reasons as to why medical cannabis interventions in primary research are often unaligned and unstandardised, including medical cannabis supply frameworks, funding limitations, and patient self-administration preferences, amongst others [4, 18].

The policy landscape concerning the use of cannabis is changing rapidly. Jurisdictions around the world have increasingly reformed their laws and regulations to permit the general adult population access to non-medical cannabis products through legal supply architectures, with an estimated 180 million people living in such jurisdictions [56]. Prevailing evidence tentatively suggests that some patients may move from accessing cannabis through medical regulatory frameworks to non-medical supply routes as the non-medical route becomes legally available [57]. To this, we highlight that the 23 systematic reviews we analysed focused on a variety of conditions that will require condition-specific clinical evidence and physician-guided treatment. For these individuals who are affected by such conditions, a primary concern will likely be accessing the most effective and safest treatments. So long as individuals, practitioners, and policymakers continue to hold an interest in the applications of medical cannabis for the treatment of a variety of conditions, the need for high-quality evidence on the applications of medical cannabis for specific conditions with an appropriate regard to the product-specific dimensions of treatment will continue into the foreseeable future.

## Conclusion

Medical cannabis is not a homogeneous group of products, but a large and complex group of substances that significantly vary in their compositions, characteristics, and potential practices of administration. Effectiveness and safety must be understood in relation to these product-specific dimensions, such that all relevant stakeholders, including patients and healthcare practitioners, hold strong knowledge of how a specific medical cannabis product may be used in treatments for a range of conditions. However, our analysis has provided evidence to suggest that systematic reviews – a highly valued form of evidence to inform medical decision-making – are frequently failing to capture how the effectiveness and safety of medical cannabis treatments vary by their type, cannabinoid composition, potency or dosage, route of administration, and frequency or length of administration. Many systematic reviews do not aim to conduct appropriate analyses to capture this information. Of those that do, many are unable to conduct such analyses for reasons primarily related to the level of variation in the characteristics, compositions, and administration of medical cannabis products used; either far too much, or far too little. This analysis highlights the need for developments in the standardisation of approaches in medical cannabis research, allowing systematic reviews to capture a granular level of information concerning the interaction of product-specific dimensions of medical cannabis, and the effectiveness and safety of such products.

## Footnotes

1 When alluding to medical cannabis products as a whole we use the expression ‘medical cannabis products’. This includes any herbal cannabis product, cannabis extract, pharmaceutical product, or synthetic cannabis product used for medical purposes, including those with and without a licensing and marketing authorization. However, given the importance of product specificity (which we discuss further in the article), where relevant and possible we provide specific details on the concrete product we are referring to in particular.

2 We note that these schemes, such as compassionate access schemes, can apply to medical cannabis products that have already received a marketing authorization.

3 The U.S. Food and Drug Administration (FDA) has approved Epidyolex and three synthetic THC products for the treatment of certain severe conditions. Readers interested in the U.S. policy context should refer to the recent overview of U.S. medical cannabis law in Hong et al., 2024.

5 From July 2024, the European Monitoring Centre for Drugs and Drug Addiction (EMCDDA) officially became the European Union Drugs Agency (EUDA).

4 For a more detailed description of the search strategy and screening process, please see Pardal et al., forthcoming [28].

## Declarations

As noted in the manuscript, this analysis was triggered by the findings of a previous study, which had been commissioned by the EMCDDA. For the current analysis, the authors did not receive support from any organization.
